# Effectiveness of Transurethral Resection of the Prostate in Managing Lower Urinary Tract Symptoms and Enhancing Quality of Life: A Prospective South Indian Study

**DOI:** 10.7759/cureus.90945

**Published:** 2025-08-25

**Authors:** Vijayanand Mani, Bhavyadeep Korrapati, Velmurugan Palaniyandi, Hariharasudhan Sekar, Sriram Krishnamoorthy

**Affiliations:** 1 Urology, Sri Ramachandra Institute of Higher Education and Research, Chennai, IND

**Keywords:** benign prostatic hyperplasia, prostate, quality of life, transurethral resection of the prostate, turp, uroflowmetry

## Abstract

Benign prostatic hyperplasia (BPH) is a non-malignant enlargement of the prostate in elderly men, commonly causing lower urinary tract symptoms (LUTS) that significantly impair quality of life (QoL). Although laser enucleation techniques are increasingly adopted, transurethral resection of the prostate (TURP) remains a widely used standard treatment. This study evaluates the clinical effectiveness of TURP in alleviating LUTS and enhancing QoL.

A prospective study was conducted at a tertiary care hospital in South India from March to August 2024. Fifty male patients aged 45-85 years, diagnosed with BPH, underwent TURP. Symptom severity and functional outcomes were assessed using the International Prostate Symptom Score (IPSS), Index of Quality of Life (IQL) score, post-void residual (PVR) urine, and uroflowmetry preoperatively and at four, eight, and 12 weeks postoperatively. Data were analysed using paired-sample t-tests.

The mean patient age was 60.7 years. Baseline IPSS was 25.1 ± 6.8, and IQL was 4.5 ± 0.9. Postoperatively, IPSS improved significantly to 16.5 ± 4.5 at four weeks and 5.8 ± 3.0 at 12 weeks. IQL remained at 4.5 ± 1.0 at four weeks but significantly improved to 1.1 ± 0.9 by 12 weeks. PVR and uroflowmetry values also showed statistically significant improvement (p < 0.001).

TURP offers substantial early and sustained relief from LUTS and significantly enhances QoL in patients with moderate to severe BPH, maintaining its relevance despite the growing use of laser techniques.

## Introduction

Benign prostatic hyperplasia (BPH) is a highly prevalent condition among ageing men, with reported incidence rates approaching 85% in certain populations [1-6]. It is primarily characterised by bladder outlet obstruction, leading to a spectrum of lower urinary tract symptoms (LUTS) that include both obstructive (hesitancy, poor stream, incomplete emptying) and irritative (frequency, nocturia, urgency) components [7]. While complications such as urinary tract infections, acute urinary retention, and obstructive uropathy may arise, they are comparatively uncommon. Nevertheless, the chronic and bothersome nature of LUTS can substantially impair quality of life (QoL), making timely diagnosis and management essential [8].

Over the past two decades, the surgical landscape of BPH has undergone a significant transformation. Technological advancements have propelled laser-based procedures, such as holmium laser enucleation of the prostate (HoLEP) and thulium fibre laser enucleation of the prostate (ThuFLEP), to the forefront, especially in tertiary and corporate healthcare centres. These modalities offer the promise of reduced morbidity, shorter hospital stays, and suitability for larger prostates. As a result, the adoption of laser technologies has increasingly overshadowed conventional surgical techniques in many modern urological practices.

Despite the emergence of newer minimally invasive techniques, transurethral resection of the prostate (TURP) continues to be widely considered the gold standard for surgical management of BPH, particularly due to its proven efficacy, cost-effectiveness, and accessibility in many healthcare settings [9].

The objective of this study is to prospectively evaluate the clinical effectiveness of TURP in improving symptom burden and QoL among men with BPH. In particular, we assess changes in the International Prostate Symptom Score (IPSS) and the Index of Quality of Life (IQL), which are validated, widely used, and freely available tools for assessing LUTS severity and QoL burden in BPH patients [7,8]. This study aims to provide robust evidence supporting the continued role of TURP in the surgical treatment of BPH, even amid the rising popularity of laser alternatives.

## Materials and methods

A prospective observational study was conducted at a tertiary care teaching institution in southern India over six months (March to August 2024). The study protocol was reviewed and approved by the Institutional Ethics Committee (Approval No: CSP/23/SEP/136/805), and written informed consent was obtained from all participants before enrolment in accordance with institutional ethical standards. A consecutive sampling method was employed to recruit eligible patients presenting with LUTS due to BPH during the study period. In total, 50 male patients aged between 45 and 85 years with a confirmed diagnosis of BPH were enrolled. All participants underwent monopolar TURP using a 26 Fr continuous-flow resectoscope, performed with a standardised operative protocol by a single senior urologist to ensure consistency across cases. Postoperative outcomes were systematically assessed at four, eight, and 12 weeks.

Eligible participants were male patients aged between 45 and 85 years who had a prostate volume ranging from 30 to 80 grams, as assessed by abdominal sonography. Additional inclusion requirements were the presence of moderate to severe LUTS, defined by an IPSS greater than 19, a post-void residual (PVR) urine volume exceeding 150 ml, and an IQL score above 3. Patients were excluded if they had a prior history of prostate surgery or urethral procedures, structural abnormalities such as urethral stricture or meatal stenosis, or neurological disorders that could affect bladder function, including Parkinson’s disease, cerebrovascular accident, or spinal cord injury.

All patients underwent a detailed clinical evaluation, which included a review of their medical history, a digital rectal examination (DRE), and appropriate imaging. Assessment parameters included IPSS, IQL score, PVR, and uroflowmetry with a focus on maximum urinary flow rate (Qmax). Data were systematically recorded using a structured data sheet that documented demographics, clinical findings, and investigation results. Patients with bothersome LUTS suggestive of BPH were evaluated for TURP candidacy. A thorough physical examination, including neurologic and genitourinary assessments, was performed to exclude prostate malignancy. Neurological evaluations included assessment of the bulbocavernosus reflex, perianal sensation, and anal tone.

Diagnostic investigations included (i) urine routine and culture to rule out infection, (ii) serum PSA to exclude malignancy, (iii) serum creatinine to assess renal function, and (iv) transabdominal sonography to evaluate hydronephrosis, perinephric changes, urinary calculi, bladder wall thickness and capacity, PVR, and prostate characteristics, including size and echotexture. Uroflowmetry was also conducted.

The IPSS is a validated seven-item questionnaire developed by the American Urological Association (AUA) and is publicly available for clinical and research use without licensing restrictions. It was used in this study in accordance with its free-to-use status, as outlined by the AUA guidelines. The IQL score, endorsed by the International Consensus Committee, is a single-question measure scored from 0 (delighted) to 6 (terrible), facilitating physician-patient discussion on symptom burden. All 50 patients underwent TURP under spinal anaesthesia. Follow-up evaluations at four, eight, and 12 weeks included repeat clinical assessments, IPSS, IQL, PVR, and uroflowmetry. Data were documented and analysed systematically.

Statistical analysis was performed using paired t-tests to compare baseline and 12-week follow-up scores. A P-value < 0.05 was considered statistically significant.

## Results

Table 1 presents the temporal changes in IPSS and IQL in patients undergoing TURP, assessed at baseline and four, eight, and 12 weeks postoperatively.

**Table 1:** Temporal changes in IPSS and IQL scores in patients undergoing TURP: baseline to 12-week follow-up. ^†^ P-values calculated for change from baseline to 12 weeks postoperatively using a paired t-test. IPSS: International Prostate Symptom Score; IQL: Index of Quality of Life; TURP: transurethral resection of the prostate.

Outcome measure	Baseline (pre-op)	4 weeks post-op	8 weeks post-op	12 weeks post-op	% improvement (pre to 12 weeks)	P-value^†^
IPSS	25.1 ± 6.8	16.5 ± 4.5	11.5 ± 3.4	5.8 ± 3.0	76.9%	<0.001
IQL	4.5 ± 0.9	4.5 ± 1.0	2.7 ± 0.6	1.1 ± 0.9	75.6%	<0.001

At baseline, patients demonstrated a mean IPSS of 25.1 ± 6.8, indicating severe LUTS, and a mean IQL score of 4.5 ± 0.9, reflecting a significantly impaired quality of life. Following TURP, there was a progressive and substantial reduction in both scores over time when compared with preoperative baseline values. By the 12th week, the mean IPSS had reduced to 5.8 ± 3.0 (a 76.9% decline from baseline), representing a shift to mild symptomatology. Similarly, the IQL score improved to 1.1 ± 0.9, marking a 75.6% improvement in perceived quality of life. The statistical analysis revealed that these improvements from baseline to 12 weeks were highly significant (P < 0.001) for both IPSS and IQL. This demonstrates the efficacy of TURP not only in alleviating LUTS but also in enhancing patient-reported outcomes.

These findings underscore the role of TURP as an effective intervention for symptomatic benign prostatic hyperplasia, with clinically meaningful improvements evident as early as four weeks and continuing through to 12 weeks postoperatively.

**Figure 1:**
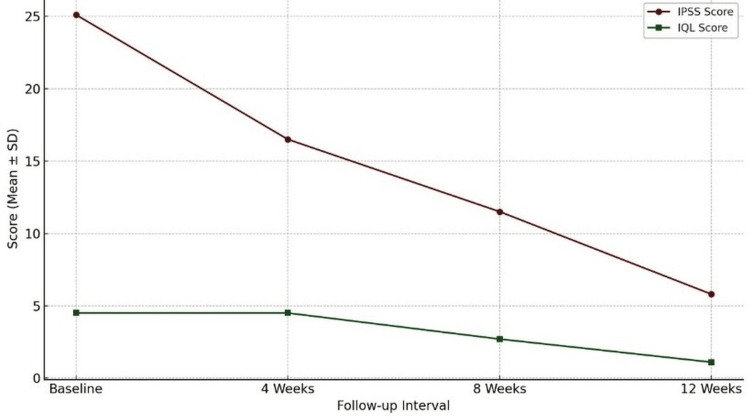
Temporal trends in symptom severity and quality of life following TURP: changes in IPSS and IQL scores over 12 weeks. IPSS: International Prostate Symptom Score; IQL: Index of Quality of Life; TURP: transurethral resection of the prostate.

Figure 1 illustrates the trend in IPSS and IQL among patients undergoing TURP, assessed at baseline and at four, eight, and 12 weeks postoperatively. The graph demonstrates a progressive and substantial decline in IPSS scores, from a baseline of 25.1 to 5.8 by week 12, reflecting significant symptomatic improvement. Similarly, IQL scores show a steady reduction, indicating enhanced patient-perceived quality of life over time. The downward trajectory of both curves highlights the early and sustained efficacy of TURP in managing LUTS and improving quality-of-life outcomes post intervention.

Table 2 presents the results of paired samples t-tests comparing pre- and post-treatment clinical parameters in patients undergoing TURP. The analysis evaluates changes in demographic, baseline clinical, and follow-up outcome measures. The mean age of patients was 60.7 years (t = 49.06, P < 0.001), reflecting the typical demographic profile of BPH presentation. Significant reductions were observed in baseline clinical parameters following TURP. Prostate size and PVR volume demonstrated marked decreases, with mean differences of 65.3 grams (t = 38.70, P < 0.001) and 225.08 ml (t = 24.57, P < 0.001), respectively. These findings affirm the anatomical and functional efficacy of the surgical intervention.

**Table 2:** Summary of paired samples t-test for pre- and post-treatment clinical parameters. PVR: post-void residual; IPSS: International Prostate Symptom Score; IQL: Index of Quality of Life.

Parameter	Mean difference	t-value	df	95% CI of the difference	P-value
Demographic					
Age (years)	60.70	49.060	49	58.21 – 63.19	<0.001
Baseline clinical measures					
Prostate size (g)	65.30	38.697	49	61.91 – 68.69	<0.001
PVR (ml)	225.08	24.573	49	206.67 – 243.49	<0.001
Pre-IPSS	25.12	23.101	49	23.19 – 27.05	<0.001
Pre-IQL	4.50	33.390	49	4.23 – 4.77	<0.001
Postoperative follow-up outcomes					
IPSS – 4 weeks	16.50	25.846	49	15.22 – 17.78	<0.001
IPSS – 8 weeks	11.52	23.667	49	10.54 – 12.50	<0.001
IPSS – 12 weeks	5.82	13.297	49	4.94 – 6.70	<0.001
IQL – 4 weeks	4.52	31.489	49	4.23 – 4.81	<0.001
IQL – 8 weeks	2.76	29.727	49	2.57 – 2.95	<0.001
IQL – 12 weeks	1.10	7.985	49	0.82 – 1.38	<0.001

Preoperative symptom burden, as measured by the IPSS and the IQL, also showed highly significant reductions. IPSS decreased by 25.12 points (t = 23.10, P < 0.001), and IQL by 4.5 points (t = 33.39, P < 0.001). Postoperatively, improvements were sustained and progressive. IPSS showed consistent declines at four weeks (16.5 points), eight weeks (11.52 points), and 12 weeks (5.82 points), all with P < 0.001. Similarly, IQL scores improved steadily across follow-ups, with reductions of 4.52, 2.76, and 1.10 points, respectively. All comparisons yielded statistically significant P-values (<0.001), underscoring the robustness of the intervention across both objective clinical metrics and patient-reported outcomes. The degrees of freedom (df = 49) correspond to the 50 paired observations analysed (df = n - 1 for paired t-tests).

**Figure 2:**
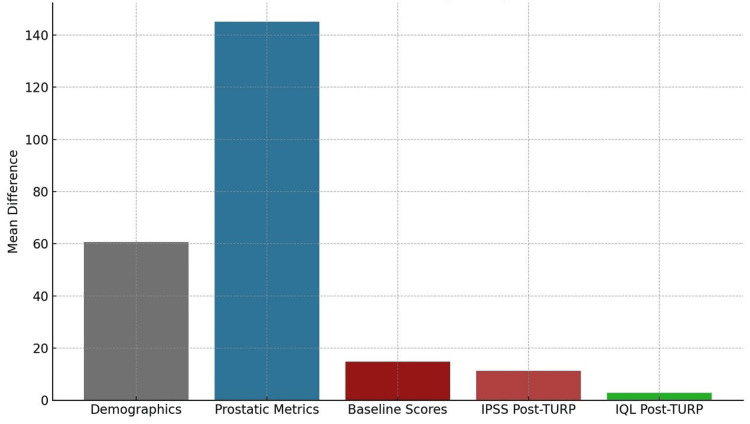
Comparative analysis of mean differences in clinical parameters before and after TURP grouped by clinical category. IPSS: International Prostate Symptom Score; IQL: Index of Quality of Life; TURP: transurethral resection of the prostate.

Figure 2 presents a consolidated bar chart illustrating the mean differences across key clinical categories before and after TURP. The demographic profile showed a mean patient age of 60.7 years, aligning with the typical age range for clinically significant BPH. In the domain of prostatic metrics, TURP resulted in substantial reductions in both prostate size and PVR urine volume, indicating clear anatomical and functional improvements. Baseline scores for the IPSS and IQL reflected a high symptom burden and markedly impaired quality of life before surgery. Following TURP, IPSS scores decreased progressively across each follow-up interval, reflecting sustained relief from urinary symptoms. Correspondingly, IQL scores improved consistently, underscoring the positive patient-reported outcomes and the overall effectiveness of the procedure in enhancing quality of life.

Table 3 summarises the results of paired samples t-tests comparing preoperative IPSS and IQL scores with those obtained at four, eight, and 12 weeks following TURP.

**Table 3:** Paired samples t-test comparing preoperative and postoperative IPSS and IQL scores. IPSS: International Prostate Symptom Score; IQL: Index of Quality of Life; SE: standard error.

Comparison	Mean difference	SD	SE mean	95% CI (lower-upper)	t-value	df	P-value
IPSS scores							
Pre-IPSS vs. IPSS – 4 weeks	8.62	8.15	1.15	6.30 – 10.94	7.48	49	<0.001
Pre-IPSS vs. IPSS – 8 weeks	13.60	7.71	1.09	11.41 – 15.79	12.48	49	<0.001
Pre-IPSS vs. IPSS – 12 weeks	19.30	7.49	1.06	17.17 – 21.43	18.23	49	<0.001
IQL scores							
Pre-IQL vs. IQL – 4 weeks	2.52	1.18	0.17	2.18 – 2.86	15.07	49	<0.001
Pre-IQL vs. IQL – 8 weeks	1.74	1.14	0.16	1.42 – 2.06	10.80	49	<0.001
Pre-IQL vs. IQL – 12 weeks	3.40	1.39	0.20	3.01 – 3.79	17.36	49	<0.001

The analysis demonstrates a consistent and statistically significant reduction in both symptom severity and QoL burden across all postoperative time points. The mean difference in IPSS progressively increased over time, from 8.62 at four weeks to 19.30 at 12 weeks, highlighting a sustained improvement in LUTS. These differences were statistically significant, with t-values ranging from 7.48 to 18.23 (P < 0.001 for all comparisons).

Similarly, IQL scores showed significant reductions, indicating enhanced patient-reported quality of life. The mean improvement in IQL was 2.52 at four weeks, 1.74 at eight weeks, and 3.40 at 12 weeks, with corresponding t-values ranging from 10.80 to 17.36 (P < 0.001). These findings confirm the clinical efficacy of TURP in not only alleviating obstructive urinary symptoms but also in significantly improving the overall quality of life for patients.

A statistical comparison of preoperative versus postoperative scores at each follow-up interval reveals significant reductions in both IPSS and IQL scores following TURP. All comparisons yielded P-values < 0.001, confirming that the observed improvements were highly significant. Notably, symptom relief and quality-of-life improvements were progressive across time points. The consistent statistical significance (P < 0.001) across all comparisons underscores the robustness and reliability of the observed outcomes.

**Figure 3:**
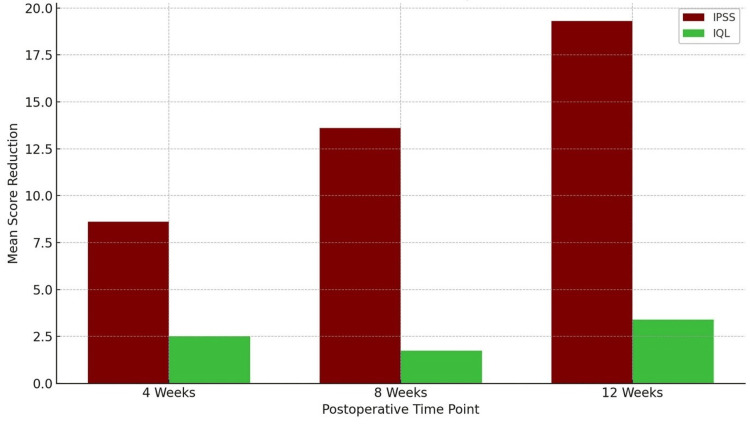
Mean reduction in IPSS and IQL scores from baseline at four, eight, and 12 weeks post TURP. IPSS: International Prostate Symptom Score; IQL: Index of Quality of Life; TURP: transurethral resection of the prostate.

Figure 3 illustrates the mean reduction in IPSS and IQL from the preoperative baseline at four, eight, and 12 weeks following TURP. The trend demonstrates a progressive and substantial improvement in both symptom severity and patient-reported quality of life over time. Notably, IPSS scores (depicted in dark red) show a steeper and more rapid decline, reflecting early and marked symptomatic relief. In contrast, IQL scores (represented in lime green) exhibit a more gradual but consistent improvement, indicating ongoing enhancement in the perceived quality of life during the postoperative period.

## Discussion

This prospective observational study confirms the clinical effectiveness of TURP in alleviating LUTS and enhancing QoL in patients with BPH, demonstrating significant improvements relative to preoperative baseline values rather than in comparison to alternative surgical modalities. Among the 50 men studied, aged 45-85 years, statistically and clinically significant reductions in symptom severity and QoL burden were noted within 12 weeks following surgery. These findings reinforce the continued relevance of TURP in the evolving landscape of surgical treatment for BPH management.

At baseline, patients demonstrated a mean IPSS of 25.1 ± 6.8 and an IQL of 4.5 ± 0.9, consistent with severe LUTS and a corresponding impairment in quality of life. Postoperative assessments revealed a progressive decline in IPSS to 5.8 ± 3.0 and an improvement in IQL to 1.1 ± 0.9 by 12 weeks, corresponding to 76.9% and 75.6% improvements, respectively (P < 0.001). These outcomes validate the robust and reproducible therapeutic benefit of TURP.

The temporal trajectory of recovery reveals rapid symptom relief in the first month, with continued QoL gains through weeks eight and 12. These findings align with prior studies by Kang et al. [10] and Hakenberg et al. [11], who reported improvements in IPSS of approximately 10.9 points at three months post TURP. Similarly, Kim et al. [12] confirmed in a systematic review that TURP yields durable reductions in the IPSS and consistent functional improvements.

Anatomical outcomes in our study also underscore the mechanical efficacy of TURP. Mean reductions of 65.3 grams in prostate volume and 225.08 ml in PVR urine were recorded (P < 0.001). Although Qmax was not part of our dataset, previous literature widely reports substantial improvements in urinary flow post TURP, further validating decompression of bladder outlet obstruction [13,14].

A particularly notable observation is the temporal dissociation between improvements in IPSS and IQL. While symptom scores declined rapidly by week four, perceptible improvements in QoL became more pronounced between weeks eight and 12. This delayed trajectory is consistent with findings by Mishriki et al. [15], who documented sustained improvements in QoL over an extended follow-up period. The implication for clinical practice is clear: patients must be appropriately counselled that while symptomatic relief is evident early on, complete restoration of quality of life may require additional recovery time.

Our data also address the ongoing debate regarding the benefit of TURP in patients with moderate LUTS. Fowler previously argued that only patients with severe baseline symptoms experience significant improvements in QoL. However, our findings suggest substantial improvements even in the presence of moderate symptom burdens, supporting a broader surgical candidacy and therapeutic utility for TURP [16].

Age did not influence outcomes. The mean age of 60.7 years aligns with epidemiological trends in BPH. Outcomes were consistent across older and younger subgroups, supporting earlier reports by Milicevic et al. [17] that age is not a limiting factor for surgical success. TURP remains safe and effective across a broad age spectrum.

**Figure 4:**
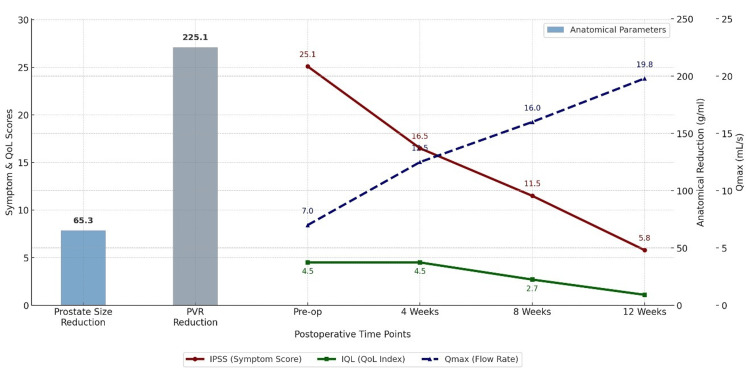
Matplotlib-based multiaxial visualization of temporal trends in IPSS, IQL, Qmax, and anatomical parameter reductions following TURP. IPSS: International Prostate Symptom Score; IQL: Index of Quality of Life; TURP: transurethral resection of the prostate; Qmax: maximum urinary flow rate; PVR: post-void residual.

Figure 4 presents a comprehensive visualisation that integrates symptom trends (IPSS), patient-reported IQL, functional flow (Qmax), and anatomical changes (prostate size and PVR). IPSS declined from 25.1 to 5.8, while IQL improved from 4.5 to 1.1. A steady increase in Qmax, from 7.0 to 19.8 mL/s, further highlights the flow restoration. Reductions in prostate size and PVR were notable, supporting mechanical relief of obstruction. This multidimensional chart illustrates the layered benefits of TURP, from symptomatic relief to functional recovery and structural decompression, and can serve as a valuable educational and clinical decision-making tool.

Table 3 and Figure 3 underscore the statistical robustness of our outcomes. The most significant symptomatic gains (mean IPSS difference: 5.7) occurred between weeks eight and 12. The greatest QoL improvements (mean IQL difference: 3.4) also emerged during this window. These patterns mirror those reported by Yang et al. [18], who found parallel improvements in symptoms and Qmax at three months.

From a clinical perspective, this evidence has practical implications. While many men initially opt for medical therapy, such as α-blockers or 5α-reductase inhibitors, these agents are often associated with side effects like sexual dysfunction, hypotension, and limited efficacy. As highlighted by Yoosuf et al. [19], many patients ultimately require surgery. In contrast, TURP offers prompt, significant, and sustained outcomes, supporting earlier surgical referrals in appropriate cases.

Minimally invasive alternatives such as prostatic urethral lift (PUL) and Rezum therapy offer shorter recovery and reduced morbidity but often show higher recurrence and retreatment rates. As Elterman et al. [20] observed, these techniques may not offer the same durability as TURP, particularly in prostates ranging from 30 to 80 grams, the size bracket evaluated in this study.

Although laser-based approaches, such as HoLEP and ThuFLEP, are gaining traction in advanced centres, TURP remains highly relevant, especially in resource-constrained environments. Its well-standardised technique, established safety, and cost-effectiveness make it a cornerstone procedure in the surgical management of BPH. Our findings strongly support the role of TURP as the gold-standard surgical intervention for moderate to severe BPH. Improvements in symptom burden, quality of life, anatomical parameters, and functional outcomes were consistent, statistically significant, and clinically meaningful. These results support the continued use of TURP across diverse patient profiles and healthcare settings.

This study has several limitations. The 12-week follow-up period, while adequate for capturing early outcomes, precludes assessment of long-term efficacy, recurrence, or late complications. The absence of a comparator group (such as medical therapy or laser procedures) limits conclusions regarding non-inferiority or equivalence. Our findings should be interpreted as evidence of TURP’s effectiveness rather than direct comparative efficacy. As a single-centre study in a tertiary care setting, generalisability to other populations and clinical environments may be limited. Additionally, selection bias may exist due to the inclusion of only patients with moderate to severe LUTS and prostate volumes between 30 and 80 grams. Postoperative sexual function, an important patient-reported outcome, was not evaluated. Lastly, although improvements in voiding parameters were observed, detailed statistical analysis of urodynamic measures such as Qmax was not performed, limiting functional correlation.

## Conclusions

This prospective study reaffirms the enduring value of TURP as a cornerstone intervention for BPH. The procedure demonstrated substantial and sustained improvements in symptom burden and patient-reported quality of life, alongside significant reductions in prostate volume and PVR urine. These outcomes highlight TURP’s dual efficacy in addressing both anatomical and functional aspects of BPH across a diverse patient population. The observed pattern of early symptom relief followed by progressive quality-of-life enhancement underscores the importance of comprehensive patient counselling regarding the expected recovery trajectory. Despite the rise of minimally invasive and laser-based alternatives, TURP remains a highly effective, accessible, and cost-efficient option, particularly in resource-constrained settings. The improvements observed in symptom burden and quality of life are relative to baseline preoperative status, not in direct comparison with other surgical or minimally invasive alternatives.
